# Molecular characteristics and virulence gene profiles of *Staphylococcus aureus* isolates in Hainan, China

**DOI:** 10.1186/s12879-019-4547-5

**Published:** 2019-10-22

**Authors:** Xuehan Li, Tao Huang, Kai Xu, Chenglin Li, Yirong Li

**Affiliations:** 1 Department of Laboratory Medicine, Zhongnan Hospital, Wuhan University, 169 Donghu Road, Wuhan, 430071 People’s Republic of China; 2 Department of Laboratory Medicine, Hainan General Hospital, Haikou, China; 3 Center of Laboratory Medicine, National Center for Cardiovascular Diseases & Fuwai Hospital, Peking Union Medical College & Chinese Academy of Medical Sciences, Beijing, China

**Keywords:** *Staphylococcus aureus*, Virulence gene, Molecular characterization, Antimicrobial susceptibility test

## Abstract

**Background:**

There have been no reports regarding the molecular characteristics, virulence features, and antibiotic resistance profiles of *Staphylococcus aureus* (*S. aureus*) from Hainan, the southernmost province of China.

**Methods:**

Two hundred twenty-seven *S. aureus* isolates, consisting of 76 methicillin-resistant *S. aureus* (MRSA) and 151 methicillin-susceptible *S. aureus* (MSSA), were collected in 2013–2014 and 2018–2019 in Hainan, and investigated for their molecular characteristics, virulence genes, antibiotic resistance profiles and main antibiotic resistance genes.

**Results:**

Forty sequence types (STs) including three new STs (ST5489, ST5492 and ST5493), and 79 Staphylococcal protein A (*spa*) types were identified based on multilocus sequence typing (MLST) and *spa* typing, respectively. ST398 (14.1%, 32/227) was found to be the most prevalent, and the prevalence of ST398-MSSA increased significantly from 2013 to 2014 (5.5%, 5/91) to 2018–2019 (18.4%, 25/136). Seventy-six MRSA isolates were subject to staphylococcus chromosomal cassette *mec* (SCC*mec*) typing. SCC*mec-*IVa was the predominant SCC*mec* type, and specifically, ST45-SCC*mec* IVa, an infrequent type in mainland China, was predominant in *S. aureus* from Hainan. The antibiotic resistance profiles and antibiotic resistance genes of *S. aureus* show distinctive features in Hainan. The resistant rates of the MRSA isolates to a variety of antibiotics were significantly higher than those of the MSSA isolates. The predominant erythromycin and tetracycline resistance genes were *ermC* (90.1%, 100/111) and *tetK* (91.8%, 78/85), respectively. Eleven virulence genes, including the Panton-Valentine leukocidin (*pvl*) and *eta*, were determined, and the frequency of *eta* and *pvl* were found to be 57.3 and 47.6%. Such high prevalence has never been seen in mainland China before.

**Conclusion:**

*S. aureus* isolates in Hainan have unique molecular characteristics, virulence gene and antibiotic resistance profiles, and main antibiotic resistance genes which may be associated with the special geographical location of Hainan and local trends in antibiotic use.

## Background

*Staphylococcus aureus* (*S. aureus*) is an important Gram-positive pathogen that causes various infectious diseases including pneumoniae and bacteremia. A previous study showed that patients with *S. aureus* infections had an excess one-year mortality of 20.2% compared with matched uninfected inpatients [1]. The genotype of *S. aureus* has been reported to influence the complications, severity, and mortality of infection. One study showed that the strains clonal complex 5 (CC5) and CC30 exhibited a significant trend toward increasing levels of hematogenous complications [2]. Another study found that patients with *S. aureus* sequence type 121 (ST121) infections often required longer hospitalization and prolonged antimicrobial therapy [3], whereas bloodstream infections by CC398, a methicillin-susceptible *Staphylococcus aureus* (MSSA), were associated with high mortality [4]. Therefore, analysis of the molecular characteristics and virulence gene profiles of *S. aureus* is important for prognosis of infection.

The molecular characteristics of *S. aureus* vary with region. In many Asian countries including China and Thailand, ST239 has been found to be the most prevalent type [5–8], whereas in the United States, ST8 (USA300) and ST121 are the most frequently observed [3, 9]. Even within China, the molecular characteristics of *S. aureus* isolates differ among cities; the predominant types in Wenzhou are ST188 and ST7 [10], the major type in Dalian and Shenyang is ST5 [11], and in Chengdu, ST59 is prevalent [12]. The molecular characteristics of *S. aureus* are also reported vary over time. Since 2000, ST239-t030-SCC*mec*III has rapidly replaced ST239-t037-SCC*mec*III as the major clone of *S. aureus* isolates in Chinese tertiary hospital care [2], whereas ST239-t030-MRSA, which in 2013 was the predominant genotype among all methicillin-resistant *S. aureus* (MRSA) strains in China, had been replaced by ST59-t437-MRSA by 2016 [13]. In addition, it was reported that the predominant clones, ST239-t030 and ST239-t037, were being replaced by the continually growing ST5-t2460 clone in 2017 in Shanghai [14]. Therefore, when monitoring the molecular characteristics of *S. aureus* isolates, it is preferable to focus on a specific region of interest at a particular time.

Hainan, the southernmost province of China, is surrounded by the South China Sea, and has a uniquely tropical monsoon and marine climate that differs significantly from that on the mainland. The island has been called a “natural large greenhouse,” and the hot and humid climate is conducive to bacterial growth. Studies of the molecular characteristics and antibiotic resistance profiles of *S. aureus* isolates from China have been carried out over the last 10 years in provinces such as Zhejiang, Guangdong, and Guangxi [15–18]. To date, however, no study has focused on the molecular characteristics and virulence gene profiles of *S. aureus* isolates in Hainan, and no hospital in Hainan has been included in any multicenter studies concerned with those characteristics of *S. aureus* in China [13, 19, 20]. Not even the CHINET surveillance system includes any hospital from Hainan. Although the total area of Hainan is relatively small, its population has now reached 10 million, and moreover, its tropical monsoon and marine climate is unique in China. These are important motivations to investigate the molecular characteristics, virulence genes, and antibiotic resistance profiles of *S. aureus* isolates from Hainan.

## Methods

### *S. aureus* isolates and primers

A total of 227 consecutive and non-duplicate *S. aureus* isolates were collected from three hospitals in 2013–2014 (*n* = 91) and 2018–2019 (*n* = 136). Of the three hospitals in Haikou city, Hainan General Hospital is a large teaching hospital with more than 100,000 admissions per year in Xiuying district; Haikou People’s Hospital is a medium-sized teaching hospital with about 50,000 admissions per year in Meinan district; and First Hospital Affiliated to Hainan Medical College is a medium-sized teaching hospital with 50,000 admissions per year in Longhua district. These isolates were collected from inpatients who had cough, fever and other clinical symptoms related to infection and whose peripheral white blood cell and/or neutrophil counts were elevated. These isolates were derived from diverse clinical specimens, including cutaneous abscess and wound secretion (*n* = 110, 48.5%), sputum and pharynx swabs (*n* = 48, 21.1%), blood (*n* = 42, 18.5%), and others (catheter tip, marrow, pleural fluid, cerebrospinal fluid, cystic cavity fluid, drainage liquid, ascites, joint fluid, biopsy, and urine) (*n* = 27, 11.9%). Only the first positive culture in the course of infection was included for further analysis. These isolates were identified by conventional microbiological methods including Gram staining, catalase, and coagulase tests, and confirmed with a VITEK 2 Compact system and a VITEK 2 AST-GP67 Test Kit (bioMerieux, Inc., Durham, NC, USA). All isolates were stored at − 80 °C for further experiments. All primers used in this study were synthesized by Tianyihuiyuan (China) (Table 1). This study was approved by the Ethics Committee of Hainan General Hospital. This was a retrospective study that did not involve collection of clinical and personal information from patients, so informed consent was not required.

**Table 1:** Primers used in this study, and the results of SCC*mec* types I–V

Primer	Nucleotide sequence(5′-3′)	Target gene	Amplicon size(*bp*)	SCC*mec* type								
				I	II	III	IV	IVa	IVb	IVc	IVd	V
β	ATTGCCTTGATAATAGCCYTCT	*ccrA2-B*	937		X		X					
a3	TAAAGGCATCAATGCACAAACACT											
ccrCF	CGTCTATTACAAGATGTTAAGGATAAT	*ccrC*	518			X						X
ccrCR	CCTTTATAGACTGGATTATTCAAAATAT											
1272F1	GCCACTCATAACATATGGAA	*IS1272*	415	X			X					
1272R1	CATCCGAGTGAAACCCAAA											
5R*mecA*	TATACCAAACCCGACAACTAC	*mecA–IS431*	359									X
5R431	CGGCTACAGTGATAACATCC											
Type IVa-F	GCCTTATTCGAAGAAACCG	*–*	776					X				
Type IVa-R	CTACTCTTCTGAAAAGCGTCG											
Type IVb-F	TCTGGAATTACTTCAGCTGC	*–*	493						X			
Type IVb-R	AAACAATATTGCTCTCCCTC											
Type IVc-F	ACAATATTTGTATTATCGGAGAGC	*–*	200							X		
Type IVc-R	TTGGTATGAGGTATTGCTGG											
Type IVd-F	CTCAAAATACGGACCCCAATACA	*–*	881								X	
Type IVd-R	TGCTCCAGTAATTGCTAAAG											
Spa-1113f	TAAAGACGATCCTTCGGTGAGC	*spa*	–									
Spa-1514r	CAGCAGTAGTGCCGTTTGCTT											
arcC-F	TTGATTCACCAGCGCGTATTGTC	*arcC*	456									
arcC-R	AGG TATCTGCTTCAATCAGCG											
aroE-F	ATCGGAAATCCTATTTCACATTC	*aroE*	456									
aroE-R	GGTGTTGTATTAATAACGATATC											
glpF-F	CTAGGAACTGCAATCTTAATCC	*glpF*	465									
glpF-R	TGGTAAAATCGCATGTCCAATTC											
gmk-F	ATCGTTTTATCGGGACCATC	*gmk*	417									
gmk-R	TCATTAACTACAACGTAATCGTA											
pta-F	GTTAAAATCGTATTACCTGAAGG	*pta*	474									
pta-R	GACCCTTTTGTTGAAAAGCTTAA											
tpi-F	TCGTTCATTCTGAACGTCGTGAA	*tpi*	402									
tpi-R	TTTGCACCTTCTAACAATTGTAC											
yqiL-F	CAGCATACAGGACACCTATTGGC	*yqiL*	516									
yqiL-R	CGTTGAGGAATCGATACTGGAAC											
PVL-F	ATCATTAGGTAAAATGTCTGGACATGATCCA	*pvl*	433									
PVL-R	GCATCAASTGTATTGGATAGCAAAAGC											
FnbA-F	GTGAAGTTTTAGAAGGTGGAAAGATTAG	*fnbA*	643									
FnbA-R	GCTCTTGTAAGACCATTTTTCTTCAC											
FnbB-F	GTAACAGCTAATGGTCGAATTGATACT	*fnbB*	524									
FnbB-R	CAAGTTCGATAGGAGTACTATGTTC											
Hla-F	CTGATTACTATCCAAGAAATTCGATTG	*hla*	209									
Hla-R	CTTTCCAGCCTACTTTTTTATCAGT											
Hlb-F	GTGCACTTACTGACAATAGTGC	*hlb*	309									
Hlb-R	GTTGATGAGTAGCTACCTTCAGT											
Sea-F	GAAAAAAGTCTGAATTGCAGGGAACA	*sea*	560									
Sea-R	CAAATAAATCGTAATTAACCGAAGGTTC											
Seb-F	ATTCTATTAAGGACACTAAGTTAGGGA	*seb*	404									
Seb-R	ATCCCGTTTCATAAGGCGAGT											
Sec-F	GTAAAGTTACAGGTGGCAAAACTTG	*sec*	297									
Sec-R	CATATCATACCAAAAAGTATTGCCGT											
eta-F	CGCTGCGGACATTCCTACATGG	*eta*	676									
eta-R	TACATGCCCGCCACTTGCTTGT											
etb-F	CAGATAAAGAGCTTTATACACACATTAC	*etb*	612									
etb-R	AGTGAACTTATCTTTCTATTGAAAAACACTC											
clfA-F	ATTGGCGTGGCTTCAGTGCT	*clfa*	292									
clfA-R	CGTTTCTTCCGTAGTTGCATTTG											
ermA-F	GTTCAAGAAC AATCAATACA GAG	*ermA*	421									
ermA-R	GGATCAGGAA AAGGACATTT TAC											
ermB-F	CCGTTTACGA AATTGGAACA GGTAAAGGGC	*ermB*	359									
ermB-R	GAATCGAGAC TTGAGTGTGC											
ermC-F	GCTAATATTG TTTAAATCGT CAATTCC	*ermC*	572									
ermC-R	GGATCAGGAA AAGGACATTT TAC											
tetM-F	AGTGGAGCGATTACAGAA	*tetM*	158									
tetM-R	CATATGTCCTGGCGTGTCTA											
tetK-F	GTAGCGACAATAGGTAATAGT	*tetK*	360									
tetK-R	GTAGTGACAATAAACCTCCTA											
tetL-F	ATAAATTGTTTCGGGTCGGTAAT	*tetL*	1077									
tetL-R	AACCAGCCAACTAATGACAATGAT											
tetO-F	AACTTAGGCATTCTGGCTCAC	*tetO*	514									
tetO-R	TCCCACTGTTCCATATCGTCA											

### Antimicrobial susceptibility testing

Antimicrobial susceptibility tests were carried out using a VITEK 2 Compact system and a VITEK 2 AST-GP67 Test Kit (bioMerieux, Inc., Durham, NC, USA). Twelve antibiotics were tested, including cefoxitin (FOX), clindamycin (CLI), erythromycin (ERY), gentamicin (GEN), levofloxacin (LEV), linezolid (LZD), oxacillin (OXA), penicillin (PEN), rifampicin (RIF), trimethoprim/sulfamethoxazole (SXT), tetracycline (TET), and vancomycin (VAN). *S. aureus* ATCC 25923 and ATCC25913 were used as the quality control strains, and the results were interpreted in accordance with Clinical and Laboratory Standards Institute (CLSI) guidelines (CLSI M100-S29) [21]. In addition, *S. aureus* isolates were further identified using PCR for amplification of *mecA* as described previously [22], in which MRSA N315 was used as the positive control strain. The *mecA*-positive and cefoxitin-resistant isolates (cefoxitin minimum inhibitory concentration ≥ 8 μg/mL) were identified as MRSA. Isolates resistant to three or more different antimicrobial classes were defined as multidrug-resistant (MDR).

### Staphylococcal protein a (spa) typing

Chromosomal DNAs were extracted from *S. aureus* isolates as described previously [23]. The extracted chromosomal DNAs were stored at − 20 °C for *spa*, *Staphylococcus* chromosomal cassette *mec* (SCC*mec*), multilocus sequence typing, and detection of virulence genes. For *spa* typing, the variable repeat region of *spa* was amplified using oligonucleotide primers [23, 24] (see Table 1) followed by sequencing. The PCR mixture and conditions were similar to those described previously [23]. The resulting amplicons were purified and subjected to Sanger dideoxy DNA sequencing (Tianyihuiyuan, China) followed by analysis using the Ridom web server (http://spaserver.ridom.de). *S. aureus* isolates that could not be classified as any known *spa* type were defined as nontypable (NT).

### Multilocus sequence typing (MLST)

MLST was carried out according to the protocol described previously [23, 25]. Seven housekeeping genes of *S. aureus*—*arcC*, *aroE*, *glpF*, *gmk*, *pta*, *tpi*, and *yqil*—were adopted for MLST. Seven respective PCR assays were conducted to amplify these seven housekeeping genes. These amplicons were sequenced using Sanger dideoxy DNA sequencing (Tianyihuiyuan, China). The resulting sequences were compared with the known alleles in the MLST database (http://saureus.mlst.net), which was used to determine ST. *S. aureus* isolates that could not be assigned to any known ST were submitted to the MLST database and assigned to new STs. The clustering of related STs, which were defined as clonal complexes (CCs), was determined using eBURST .

### Staphylococcus chromosomal cassette mec (SCCmec) typing

The MRSA isolates were subjected to SCC*mec* typing as previously described [26]. MRSA isolates with suspected SCC*mec*IV were recharacterized by additional multiplex PCR as subtypes IVa, IVb, IVc, and IVd as described by Zhang et al. [22]. MRSA isolates that could not be assigned to any above type were defined as NT. All primers are listed in Table 1.

### Detection of virulence genes and antibiotic resistance genes

Eleven virulence genes, including the Panton-Valentine leukocidin (*pvl)*, the staphylococcal enterotoxin genes (*sea*, *seb*, *sec*), the exfoliative toxin genes (*eta*, *etb*), the hemolysin genes (*hla*, *hlb*), and the adhesion factor genes (*fnbA*, *fnbB*, *clfA*) were detected using PCR assays. The PCR mixture and conditions were similar to those described previously [23]. The common ERY resistance genes (*ermA, ermB, ermC*), and the TET resistance genes (*tetL, tetK, tetM, tetO*) were examined using PCR assays as previously described [27, 28].

### Statistical analysis

Statistical analyses were performed using SPSS Statistics 24.0 for Windows. Data were analyzed using the chi-square or Fisher’s exact tests. All statistical tests were two-tailed, and *p* < 0.05 or *p* < 0.01 (Fisher’s exact tests among three groups) was considered to indicate statistical significance.

## Results

### Antimicrobial susceptibility and antibiotic resistance genes

**Fig. 1:**
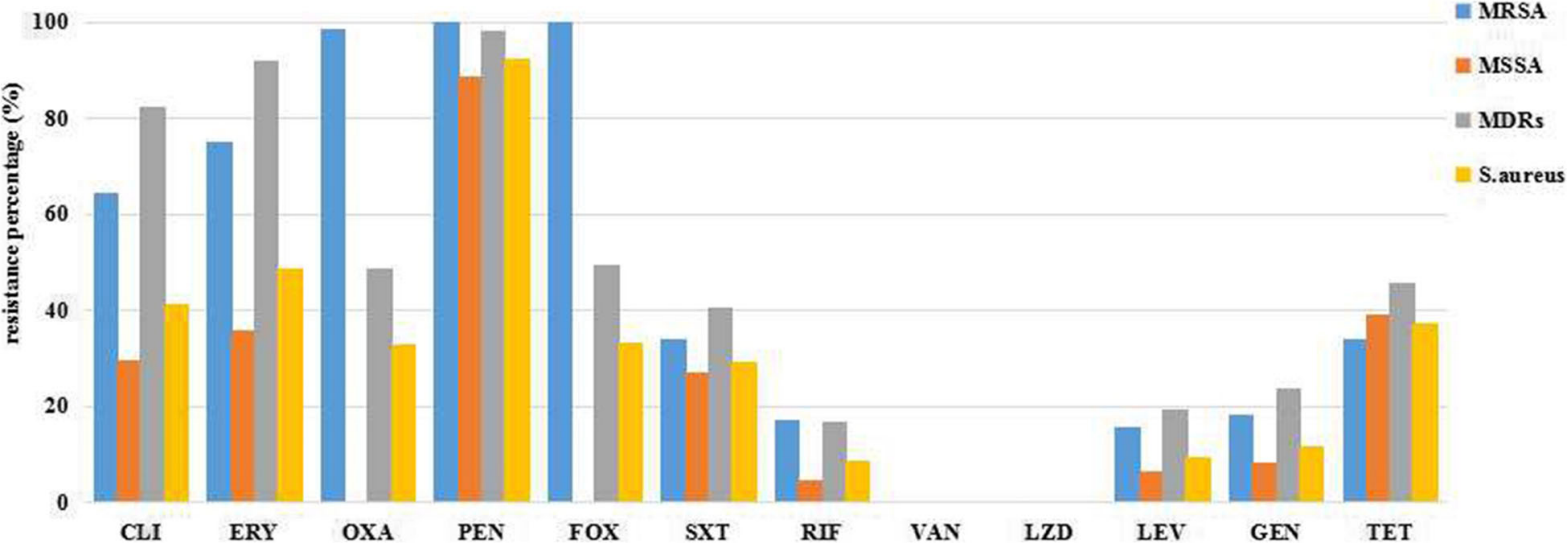
Antimicrobial resistance profiles of MRSA, MSSA, MDRs and *S. aureus* isolates

A total of 227 *S.aureus* isolates were tested for antimicrobial susceptibility. The antimicrobial resistance profiles of the *S. aureus*, MRSA, MSSA and MDR isolates are shown in Fig. 1. No *S. aureus* isolate was resistant to VAN or LZD, but most were resistant to GEN (14.1%), LEV (10.6%), and RIF (19.8%). Less than 50% of isolates were resistant to the remaining antibiotics, except for PEN, to which 92.5% had resistance. All of 76 FOX-resistant isolates, including an OXA susceptible-MRSA (OS-MRSA), were found to be *mecA*-positive, and were thus classified as MRSA isolates. Statistical analysis showed that the MRSA isolates had significantly higher resistance rates to PEN than the MSSA isolates (100.0% vs. 88.7%, *p* = 0.002), ERY (75.0% vs. 35.8%, *p* < 0.001), CLI (64.5% vs. 29.8%, *p* < 0.001), GEN (18.4% vs. 8.6%, *p* = 0.031), RIF (17.1% vs. 4.6%, *p* = 0.002), and LEV (15.8% vs. 6.6%, *p* = 0.028).

A total of 111 ERY-resistant *S. aureus* isolates were found and used to examine the presence of e*rm* gene. The most prevalent *erm* gene was *ermC* (90.1%, 100/111), followed by *ermB* (38.7%, 43/111) and *ermA* (21.6%, 24/111). In the ERY-resistant MSSA isolates, the frequencies of the three ERY-resistant genes showed the same trend as in the ERY-resistant MRSAs. *ermC* (87.0%, 47/54) was the most prevalent gene, followed by *ermB* (38.9%, 21/54) and *ermA* (11.1%, 6/54). The ERY-resistant MRSA isolates had higher frequencies of *ermA* than the ERY-resistant MSSA isolates (χ2 = 6.855, *p* < 0.05). All of 85 TET-resistant isolates carried the TET-resistant genes *tet*. The prevalences of *tetK, tetM, tetL* and *tetO* were 91.8% (78/85), 67.1% (57/85), 23.5% (20/85) and 0.0% (0/85), respectively. In the TET-resistant MSSA isolates, the most prevalent TET-resistant gene was still *tetK* (93.2%, 55/59), followed by *tetM* (59.3%, 35/59), *tetL* (23.7%, 14/59), and *tetO* (0.0%, 0/59). TET-resistant MRSA isolates had higher frequencies *tetM* than the TET-resistant MSSA isolates(χ2 = 5.227, *p* < 0.05).

One hundred thirteen (49.8%) *S. aureus* isolates were found to be MDR, defined as having resistance to more than three classes of antibiotics (Table 2). The chi-square test showed that the prevalence of MDR was significantly higher in the MRSA isolates than in the MSSA isolates (Table 2)(χ2 = 26.115, *p* < 0.05). In addition, when comparing the *S. aureus* isolates collected in 2013–2014 with those from 2018 to 2019, the resistance rates to all antibiotics except SXT were broadly similar. Compared with those collected in 2018–2019, the *S. aureus* isolates from 2013 to 2014 had a higher resistance rate to SXT (64.8% vs. 5.9%, *p* < 0.05) and a greater prevalence of MDR (61.5% vs. 41.9%, *p* < 0.05).

**Table 2:** The frequency of MDRs, main STs, and virulence genes among MRSA and MSSA

	MDRs	Main STs			Virulence genes										
isolates(n)	MDRs (n,%)	ST398 (n,%)	ST188 (n,%)	ST45 (n,%)	*pvl* (n,%)	*fnbA* (n,%)	*fnbB* (n,%)	*hla* (n,%)	*hlb* (n,%)	*sea* (n,%)	*seb* (n,%)	*sec* (n,%)	*eta* (n,%)	*etb* (n,%)	*clfA* (n,%)
MRSA(76)	56 (73.7)	2 (2.6)	1 (1.3)	20 (26.3)	31 (40.8)	36 (47.4)	31 (40.8)	74 (97.4)	51 (67.1)	18 (23.7)	38 (50.0)	38 (50.0)	47 (61.8)	15 (19.7)	76 (100.0)
MSSA(151)	57 (37.7)	30 (19.9)	29 (19.2)	3 (2.0)	77 (51.0)	51 (33.8)	82 (54.3)	150 (99.3)	110 (72.8)	17 (11.3)	70 (46.4)	25 (16.6)	83 (55.0)	28 (18.5)	151 (100.0)
*S.aureus*(227)	113 (49.8)	32 (14.1)	30 (13.2)	23 (10.1)	108 (47.6)	87 (35.7)	113 (49.8)	224 (98.7)	161 (70.9)	35 (15.4)	108 (47.6)	63 (27.8)	130 (57.3)	43 (18.9)	227 (100.0)
*p* value^a^	< 0.01	< 0.01	< 0.01	< 0.01	0.146	0.047	0.055	0.542	0.369	0.014	0.604	< 0.01	0.323	0.829	–

^a^The frequency of MDRs, main STs, and virulence genes in MRSA isolates were compared with those in MSSA isolates

### MLST, spa, and SCCmec typing

**Fig. 2:**
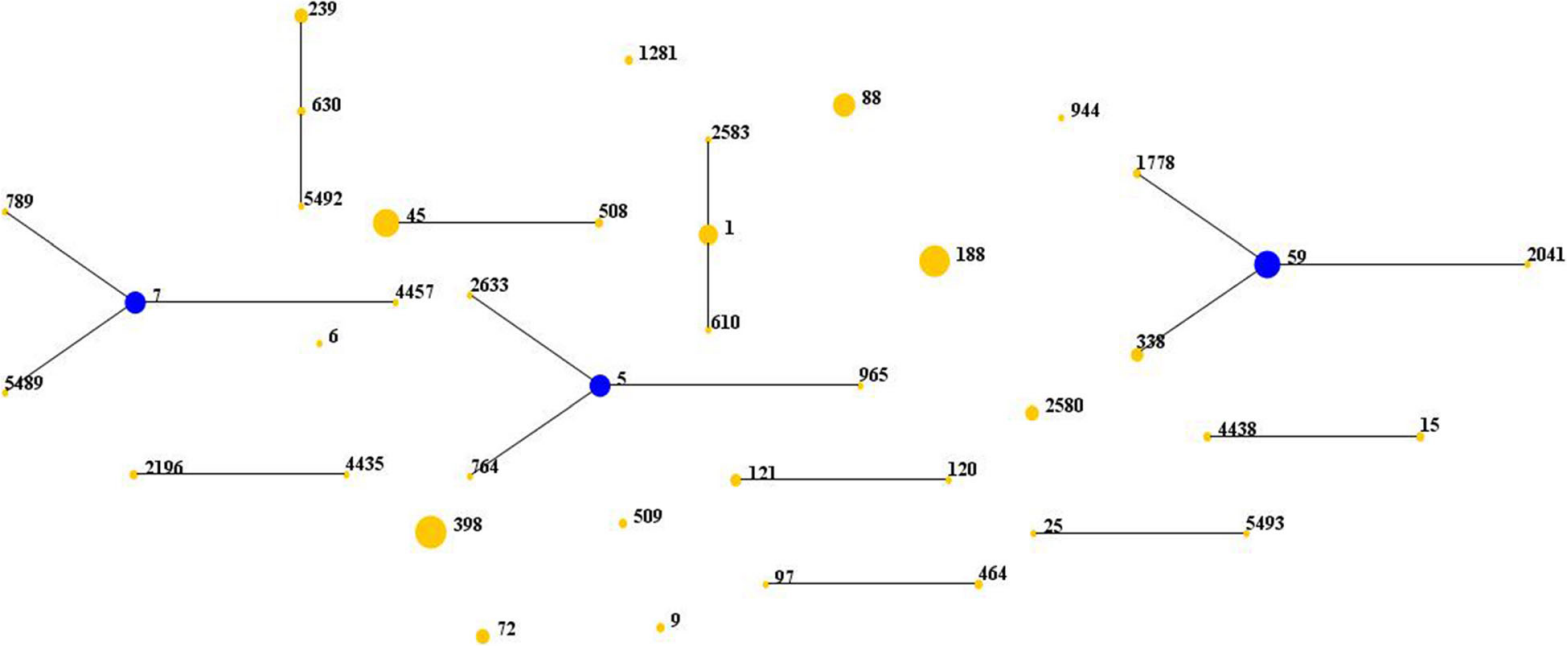
Distribution of STs in the clonal complexes. The diagram generated by eBURST based on the MLST data of this study, representing the relationships of 227 *S. aureus* isolates identified by MLST typing. Each number implies an MLST ST, STs that are linked by a line belong to the same cluster and the dot area indicates the prevalence of the ST in the MLST data of this study

Forty STs belonging to 19 CCs and 2 singletons were identified by eBURST. As shown in Table 3 and Fig. 2, ST398 (14.1%, 32/227) was the most prevalent, followed by ST188 (13.2%, 30/227) and ST45 (10.1%, 23/227). It was found that 78.1% (25/32) of the ST398 isolates, 80.0% (24/30) of the ST188 isolates, and 91.3% (21/23) of the ST45 isolates were derived from Hainan General Hospital. In addition, three isolates could not be assigned to any known ST, so these novel alleles were submitted to the MLST database, and three new STs, ST5489, ST5492 and ST5493, were assigned. By *spa* typing, 79 *spa* types were found. The most prevalent was t189 (12.3%, 28/227), followed by t437 (7.9%, 18/227), t116 (7.5%, 17/227), and t011 (6.6%, 15/227). When the STs and *spa* typing were combined, the predominant combinations were ST188-t189 (12.3%, 28/227), ST45-t116 (7.5%, 17/227), ST59-t437 (7.0%, 16/227), ST398-t011 (6.6%, 15/227), ST398-t034 (4.8%, 11/227), and ST7-t091 (4.8%, 11/227). A strong association was observed between certain STs and *spa* types: ST188 was primarily associated with t189 (93.3%, 28/30); ST45 was associated mainly with t116 (73.9%, 17/23); and ST59 was associated mainly with t437 (72.7%, 16/22).

**Table 3:** Molecular characteristics of *S. aureus* isolates collected in this study

CC (no.)	2013–2014 (91 isolates)					2018–2019 (136 isolates)				
	MLST(no.)	*spa*(no.)	MRSA(no.)	MSSA(no.)	SCC*mec*(no.)	MLST(no.)	*spa*(no.)	MRSA(no.)	MSSA(no.)	SCC*mec*(no.)
CC398(32)	ST398(5)	t011(3)		3		ST398(27)	t011(12)		12	
		t034(2)		2			t034(9)	2	7	V(2)
							t1451(3)		3	
							t571(1)		1	
							t1580 (1)		1	
							NT(1)		1	
CC59(30)	ST59(8)	t437(4)	1	3	IVa(1)	ST59(14)	t437(12)	9		IVa(5), V(4)
		t441(1)	1		V(1)		t3385(1)	1		IVa(1)
		t1212(1)		1			t5795(1)	1		IVa(1)
		t2356(1)	1		IVa(1)	ST338(3)	t437(1)		1	
		t3592(1)	1		V(1)		t1751(2)	2		V(1),NT(1)
	ST338(2)	t1751(2)		2						
	ST1778(2)	t437(1)		1		ST2041(1)	t13874(1)		1	
		t2365(1)	1		IVa(1)					
CC188(30)	ST188(13)	t189(12)		12		ST188(17)	t189(16)	1	15	IVa(1)
		t4950(1)		1			t2174(1)		1	
CC45(25)	ST45(13)	t116(10)	8	2	IVa(8)	ST45(10)	t116(7)	6	1	IVa(6)
		t015(1)	1		IVa(1)		t026(1)	1		IVa(1)
		t2131(1)	1		IVa(1)		t157(1)	1		IVa(1)
		NT(1)	1		IVa(1)		t3349(1)	1		IVa(1)
						ST508(2)	t1203(1)	1		NT(1)
							t908(1)	1		IVa(1)
CC5(17)	ST5(6)	t002(3)		3		ST5(8)	t2358(2)	2		IVa(2)
		t954(1)		1			t548(1)		1	
		t6212(1)		1			t777(1)		1	
		t2358(1)	1		IVa(1)		t1265(1)		1	
	ST965(1)	t062(1)	1		IVa(1)		t179(1)		1	
							t2980(1)		1	
							t9987(1)		1	
						ST764(1)	t1084(1)	1		II(1)
						ST2633(1)	t010(1)		1	
CC7(17)	ST7(4)	t091(4)		4		ST7(10)	t091(7)		7	
							t867(1)		1	
							t2874(1)		1	
							t3932(1)		1	
	ST5489(1)	t091(1)		1		ST789(1)	t2453(1)		1	
	ST4457(1)	t796(1)		1						
CC88(16)	ST88(8)	t1376(4)	1	3	II(1)	ST88(8)	t1376(3)	1	2	IVa(1)
		t2592(1)	1		IVa(1)		t4333(2)		2	
		t3622(1)		1			NT(3)		3	
		t15796(1)		1						
		NT(1)		1						
CC1(14)	ST1(4)	t127(1)		1		ST1(8)	t127(5)	1	4	NT(1)
		t2207(3)	3		NT(3)		t2207(2)	2		NT(2)
	ST610(1)	t2207(1)	1		II(1)		t114(1)		1	
						ST2583(1)	t1381(1)	1		IVa(1)
CC8(9)	ST239(3)	t030(2)	2		III(2)	ST239(3)	t030(2)	2		III(2)
		t037(1)	1		III(1)		t037(1)	1		III(1)
						ST630(2)	t377(1)		1	
							t4549(1)		1	
						ST5492(1)	t1987(1)		1	
CC2580(6)	ST2580(5)	t3351(4)	4		IVa(1), IVc(3)	ST2580(1)	t3351(1)	1		IVc(1)
		t4875(1)	1		IVc(1)					
CC72(6)	ST72(2)	t148(2)		2		ST72(4)	t148(3)		3	
							t3092(1)		1	
CC121(5)	ST121(4)	t269(1)		1		ST120(1)	t2613(1)	1		NT(1)
		t162(2)		2						
		t159(1)		1						
CC15(4)	ST15(1)	t1492(1)		1		ST15(1)	t085(1)		1	
	ST4438(2)	t084(2)		2						
CC97(3)	ST464(1)	t3992(1)		1		ST97(1)	t267(1)		1	
						ST464(1)	t3904(1)		1	
CC2196(3)	ST4435(1)	t037(1)	1		IVa(1)	ST2196(2)	NT(2)		2	
CC9(2)	ST9(1)	t899(1)		1		ST9(1)	t899(1)	1		I(1)
CC509(2)						ST509(2)	t375(2)	1	1	IVa(1)
CC1281(2)						ST1281(2)	t164(2)		2	
CC25(2)	ST5493(1)	t12584(1)		1		ST25(1)	t280(1)		1	
Singletons(2)	ST6(1)	t304(1)	1		IVa(1)					
	ST944(1)	t616(1)		1						

*NT* Non-typeable

The major types of *S. aureus* collected in 2013–2014 were ST188 (14.3%), ST45 (14.3%), ST59 (8.8%), and ST88 (8.8%), whereas in 2018–2019, ST398 (19.9%), ST188 (12.5%), ST59 (10.3%), ST45 (7.4%), and ST7 (7.4%) were the top five types. Among the STs that exhibited OXA sensitivity, the two predominant types in 2013–2014 were ST188-MSSA (14.3%) and ST45-MRSA (12.1%), whereas in 2018–2019 they were ST398-MSSA (18.4%) and ST59-MRSA (8.1%). The prevalence of ST398-MSSA markedly increased from 2013 to 2014 (5.5%) to 2018–2019 (18.4%), and this increase was significant (*p* < 0.05).

Among the 76 MRSA isolates, 6 SCC*mec* types or subtypes, namely types I, II, III, IVa, IVc, and V, were found. The most common SCC*mec* type was IVa, which was found in 43 isolates (56.6%, 43/76), whereas types I, II, III, IVc, and V were found in 1, 3, 6, 5, and 9 isolates, respectively. Nine isolates, including OS-MRSA, were classified as NT for SCC*mec* typing. When the STs and SCC*mec* typing were combined, the predominant combination was ST45-SCC*mec* IVa (8.8%, 20/227), and no significant difference was found in the positive rate of ST45-SCC*mec* IVa between the *S. aureus* isolates collected in 2013–2014 and 2018–2019 (12.1% vs. 6.6%, *p >* 0.05) **(**Table 3**)**.

### Virulence gene profiles

The frequencies of the virulence genes identified in the 227 *S. aureus* isolates are listed in Table 4. *ClfA* was present in all *S. aureus* isolates, *hla*, *hlb*, and *eta* were detected in 98.7, 70.9, and 57.3% of these isolates, respectively, whereas the remaining ones were found in less than 50%. One hundred and twenty (52.9%) *S. aureus* isolates harbored six or more virulence genes. Of those 120 isolates, 11 contained 9 virulence genes, 31 had 8 such genes, 38 carried 7, and 40 carried 6. The frequencies of *fnbA*, *sea*, and *sec* were significantly higher in the MRSA isolates than in the MSSA isolates, but no significant difference was found in the likelihood of harboring six or more virulence genes between the MRSA and MSSA isolates (56.6% vs. 51.0%, *p >* 0.05). Compared with those collected in 2013–2014, the *S. aureus* isolates from 2018 to 2019 had higher frequency of *pvl*, *fnbB*, *hlb*, *seb*, *eta*, and *etb* and higher rates of harboring six or more virulence genes*.*

**Table 4:** The frequency of virulence genes among main types of *S. aureus* isolates and the comparison of two time periods

Virulence genes	*S. aureus* (*n* = 227)n(%)	ST398 (*n* = 32)n(%)	ST188 (*n* = 30)n(%)	ST45 (*n* = 23)n(%)	2013–2014 (*n* = 91)n(%)	2018–2019 (*n* = 136)n(%)	*P* value^a^
*pvl*	108 (47.6)	26 (81.3)	11 (36.7)	4 (17.4)	25 (27.5)	83 (61.0)	< 0.01
*fnbA*	87 (35.7)	7 (21.9)	7 (23.3)	10 (43.5)	33 (36.3)	54 (39.7)	0.601
*fnbB*	113 (49.8)	31 (96.9)	6 (20.0)	6 (26.1)	19 (20.9)	94 (69.1)	< 0.01
*hla*	224 (98.7)	32 (100.0)	29 (96.7)	23 (100.0)	91 (100.0)	133 (97.8)	0.405
*hlb*	161 (70.9)	20 (62.5)	19 (63.3)	6 (26.1)	44 (48.4)	117 (86.0)	< 0.01
*sea*	35 (15.4)	5 (15.6)	2 (6.7)	1 (4.3)	13 (14.3)	22 (16.2)	0.699
*seb*	108 (47.6)	11 (34.4)	18 (60.0)	8 (34.8)	35 (38.5)	73 (53.7)	0.024
*sec*	63 (27.8)	2 (6.3)	5 (16.7)	22 (95.7)	28 (30.8)	35 (25.7)	0.406
*eta*	130 (57.3)	24 (75.0)	14 (46.7)	22 (95.7)	21 (23.1)	109 (80.1)	< 0.01
*etb*	43 (18.9)	10 (31.3)	6 (20.0)	3 (13.0)	0 (0.0)	43 (31.6)	< 0.01
*clfA*	227 (100.0)	32 (100.0)	30 (100.0)	23 (100.0)	91 (100.0)	136 (100.0)	–

^a^The frequency of virulence genes of *S. aureus* isolates in 2013–2014 were compared with those in 2018–2019

### Characteristics of the major clones ST398, ST188, and ST45

The most abundant sequence type found in this study was ST398 (14.1%, 32/227) followed by ST188 (13.2%, 30/227) and ST45 (10.1%, 23/227). Most ST398 (93.8%, 30/32) and ST188 (96.7%, 29/30) isolates were MSSA, whereas most ST45 (87.0%, 20/23) isolates were MRSA and all ST45-MRSA isolates belonged to the SCC*mec* IVa type (Tables 2 and 3). The ST398 (χ^2^ = 17.685, *p* < 0.01) and ST188 isolates (*p* < 0.01) had higher resistance rates to TET than the ST45 isolates. In addition, no significant difference was seen in the resistance rate to any antibiotics between ST398, ST188 and ST45 isolates.

Of the 11 tested virulence genes, *pvl* and *fnbB* were more frequent in ST398 isolates than in ST45 (χ^2^ = 22.010 and χ^2^ = 30.457, respectively, *p* < 0.01) and ST188 isolates (χ^2^ = 12.790 and χ^2^ = 38.027, respectively, *p* < 0.01). The prevalence of *sec* in ST45 isolates was higher than that in ST398 (χ^2^ = 43.487, *p* < 0.01) and ST188 isolates (χ^2^ = 32.500, *p* < 0.01), whereas the prevalence of *eta* in ST45 isolates was higher than in ST188 isolates (χ^2^ = 14.339, *p* < 0.01). On the contrary, the positive rate of *hlb* in ST45 isolates was lower than in ST398 (χ^2^ = 7.118, *p* < 0.01) and ST188 isolates (χ^2^ = 7.248, *p* < 0.01). No significant difference was found in the positive rate of any other virulence genes between any two of the three STs (Table 4).

## Discussion

A total of 227 *S. aureus* isolates were collected in 2013–2014 and 2018–2019 from three hospitals in Hainan province for investigation of their antimicrobial resistance, virulence gene profiles, and molecular characteristics. The results showed that all isolates were susceptible to VAN and LZD, in agreement with most previous studies in mainland China [29–31]. In addition, when comparing the *S. aureus* isolates collected in 2013–2014 and 2018–2019, no significant difference was found in the resistance rates to the remaining antibiotics except that to SXT. Therefore, both sets of isolates were combined for analysis, and the average resistance rates to PEN, ERY, CLI, TET, FOX, OXA, GEN, LEV, and RIF were found to be 92.5, 48.9, 41.4, 37.4, 33.5, 33.0, 11.9, 9.7, and 8.8%, respectively. For comparison, in mainland China in the first half of 2018, the corresponding average rates were reported to be 92.7, 64.5, 38.4%, unreported, 34.4, 34.4, 18.7, 22.4, and 5.2% (www.chinets.com). While in Turkey in 2017, the average rates to OXA, RIF, VAN and LZD were 23.0, 14.0, 0.0, and 0.0%, respectively. In the United States in 2017, the average rates to OXA, ERY, RIF, VAN, and LZD were 45.0, 41.0, 1.0, 0.0, and 0.0%, respectively, whereas in Russia in 2017, they were 16.0, 2.0, 0.0, and 0.0%, and in Australia they were 19.0, 1.0, 1.0, and 0.0%, respectively (resistancemap.cddep.org/AntibioticResistance.php). The *S. aureus* isolates from Hainan had resistance rates against some antibiotics similar to those from mainland China and other countries, but differences were found in the resistance rates to ERY and LEV. In addition, the resistance rate to SXT in the *S. aureus* isolates collected in 2018–2019 (5.9%) was significantly lower than for those collected in 2013–2014 (64.8%), whereas the resistance rate to SXT has remained stable in recent years in mainland China. 10.1% in 2014 and 14.3% in the first half of 2018 (http://www.chinets.com). The steep decline in resistance to SXT may be due to the reduced use of this antibiotic in recent years in Hainan.

ERY and TET resistance depend on the presence of resistance genes *erm* and *tet,* respectively. The predominant resistance gene in the ERY-resistant isolates was *ermC*, which differed from previous studies in which most of the ERY-resistant strains harboured *ermA* [27, 32]. In this study, *ermB* was present in 38.7% of ERY-resistant isolates, whereas in most previous studies, *ermB* was rare or not detected at all [27, 32]. Therefore it is concluded that *S. aureus* isolates in Hainan have characteristic resistance genes for erythromycin resistance. Most of the TET-resistanct isolates harbored *tetM* and *tetK,* which indicates that those variants were responsible for resistance to TET, consistent with previous studies [28, 32, 33]. Our study shows that the frequency of *tetM* was higher in TET-resistant MRSAs than in TET-resistant MSSAs, consistent with the previous finding that the resistance mechanism mediated by *tetM* is predominant among TET-resistant MRSAs [34].

MLST typing, *spa* typing, and SCC*mec* typing were performed to analyze the molecular characteristics of the *S. aureus* isolates. ST398, ST188, and ST45 were the predominant STs among the *S. aureus* isolates in this study, among which ST398 and ST45 were the predominant clones in the MSSA and MRSA isolates, respectively. In addition, the most common SCC*mec* type was IVa, and ST45-SCC*mec* IVa was the most prevalent combination of ST and SCC*mec* typing in the MRSA isolates. ST188 and ST239 were previously reported as the predominant STs in MSSA and MRSA isolates, respectively [11, 19, 20, 35, 36]. Two of these studies were multicenter studies that showed that ST239-SCC*mec* III was the predominant MRSA genotype, but no ST45 clones were observed [11, 20]. A recent study in Shanghai showed that ST239-t030 and ST239-t037 were being driven out by the continual growth of the ST5-t2460 clone [14]. Therefore, it can be concluded that the molecular characteristics of *S. aureus* isolates in Hainan differ significantly from those in mainland China. It is reasonable to speculate that the divergent molecular characteristics of *S. aureus* isolates in Hainan are associated with the special geographical location of Hainan.

ST398 MSSA was found to be the most prevalent in Hainan province, and the patients with the ST398 MSSA isolates had no history of contact with livestock, confirming that the ST398 MSSA isolates we collected are of human origin. In addition, in the short span of 5 years, the prevalence of ST398 MSSA increased from 5.5 to 18.4% in Hainan. Similar to the epidemic situation in Hainan province, ST398 MSSA have been increasingly reported as a cause of invasive infections in patients without livestock contact [4]. In cohorts of patients in France, the number of ST398 MSSA cases was shown to increase from zero in 1999 to 4.6% in 2010, including 13.8% of cases with *S. aureus* bloodstream infections [4, 37]. Another retrospective study in France found that only 1.9% of bone and joint infection (BJI) MSSA strains were screened to be ST398 in 2008, whereas in 2010–2012, 14.0% of BJI MSSA strains belonged to ST398 [38]. Therefore, ST398 MSSA has emerged as an invasive pathogen causes bloodstream infections, BJIs, and potentially other conditions. Evidence suggests that ST398 MRSA and ST398 MSSA belong to distinct lineages [39]. It is well known that the ST398 MRSA lineage, associated with livestock, has become a worldwide threat within the past decade [4]. However, ST398 MSSA is a frequent source of *S. aureus* infections between individuals in households. This contrasts with the limited transmissibility of livestock-associated ST398 MRSA strains between humans [40]. ST398 MSSA has enhanced adhesion to human skin keratinocytes and keratin and it is more closely linked with human infections than ST398 MRSA. In addition, it was reported that the 30-day all-cause mortality was higher for patients with ST398 MSSA bloodstream infection than for a control group with non-ST398 MSSA infection [4]. Because ST398 MSSA has become the most prevalent ST in *S. aureus* isolates from Hainan and may be linked to higher mortality, it is necessary to monitor the changes in the molecular characteristics of *S. aureus* to prevent the wider dissemination of that strain.

The virulence factors of *S. aureus* play an important role during pathogenesis [41, 42]. Similar to the majority of studies in mainland China [8, 43], almost all strains in our study were positive for *clfA* and *hla*, confirming that these were the most common virulence factors in *S. aureus*, and no regional difference was seen in their distribution. Notably, the frequencies of *eta* and *pvl* were 57.3 and 47.6%, much higher than those in mainland China [10, 16, 19]. ST45, a common type of *S. aureus* isolate in Hainan, was found to have an *eta* prevalence of 95.7% in our results. Meanwhile, ST398, a clone with a low prevalence of *pvl* in previous studies [37, 40], had a frequency of 81.3% in this study. Together, these findings indicate that *S. aureus* isolates in Hainan have somewhat higher positive rates of *eta* and *pvl*. Previous studies reported some virulence genes are linked to specific molecular types [43, 44]. For example, ST8 (USA300) was linked to the acquisition of the enterotoxin Q and K genes. ST36 (USA200) was associated with the acquisition of the enterotoxin A gene and the toxic shock syndrome toxin 1 gene [44]. Therefore, it is rational to speculate that the higher rates of *eta* and *pvl* could be associated with the different distribution of STs. This implies that the molecular characteristics of *S. aureus* isolates affect their virulence gene profiles, leading us to conclude that *S. aureus* isolates collected in Hainan have distinct virulence gene profiles compared with those collected in mainland China. In addition, compared with the 2013–2014 isolates, the *S. aureus* isolates collected in 2018–2019 carried more virulence genes, but their rate of MDR was lower. The opposite trend between antibiotic resistance and virulence may be related to balance the energetic requirements for expressing resistance and producing toxins, which suggests that the antibiotic resistance and virulence of pathogens are in competition during evolution [45, 46]. This study has some limitations. First, the small sample size limited the broad representativeness of the study. Second, we had no information about the relationship between clinical data (e.g. mortality, severity) and molecular characteristics of the isolates, which will be the focus in further research. These fields.

## Conclusions

*S. aureus* isolates in Hainan have unique molecular characteristics and virulence gene profiles. ST398-MSSA was the most common type of MSSA isolate and ST45-SCC*mec* IVa was the predominant type of MRSA isolate, neither of which had been reported in China before. Differences were also found between the antibiotic resistance and virulence gene profiles of the ST398 and ST45 isolates. ST398-MSSA showed a clear growth trend from 2013 to 2014 to 2018–2019, which deserves attention from public health services.

## Data Availability

The datasets used and/or analysed during the current study are available from the corresponding author on reasonable request.

## Abbreviations

CC: Clonal complex
MDR: Multidrug resistance
MLST: Multilocus sequence typing
MRSA: Methicillin-resistant *Staphylococcus aureus*
MSSA: Methicillin-susceptible *Staphylococcus aureus*
OS-MRSA: Oxacillin susceptible-MRSA
PVL: Panton-Valentine leukocidin
*S. aureus*: *Staphylococcus aureus*
SCC*mec*: *Staphylococcus* chromosomal cassette *mec*
